# Inhibition of autophagy delays motoneuron degeneration and extends lifespan in a mouse model of spinal muscular atrophy

**DOI:** 10.1038/s41419-017-0086-4

**Published:** 2017-12-20

**Authors:** Antonio Piras, Lorenzo Schiaffino, Marina Boido, Valeria Valsecchi, Michela Guglielmotto, Elena De Amicis, Julien Puyal, Ana Garcera, Elena Tamagno, Rosa M Soler, Alessandro Vercelli

**Affiliations:** 1 Department of Neuroscience, University of Torino, Torino, Italy; 2 Neuroscience Institute Cavalieri Ottolenghi (NICO), University of Torino, Torino, Italy; 3 Karolinska Institutet, Dept NVS, Center for Alzheimer Research, Division for Neurogeriatrics, 141 57 Huddinge, Sweden; 4 Department of Fundamental Neurosciences, University of Lausanne, Lausanne, Switzerland; 5 Unitat de Senyalització Neuronal, Dep. Medicina Experimental, Universitat de Lleida-IRBLLEIDA, Rovira Roure 80, 25198 Lleida, Spain

## Abstract

Spinal muscular atrophy (SMA) is a recessive autosomal neuromuscular disease, due to homozygous mutations or deletions in the telomeric survival motoneuron gene 1 (*SMN1*). SMA is characterized by motor impairment, muscle atrophy, and premature death following motor neuron (MN) degeneration. Emerging evidence suggests that dysregulation of autophagy contributes to MN degeneration. We here investigated the role of autophagy in the SMNdelta7 mouse model of SMA II (intermediate form of the disease) which leads to motor impairment by postnatal day 5 (P5) and to death by P13. We first showed by immunoblots that Beclin 1 and LC3-II expression levels increased in the lumbar spinal cord of the SMA pups. Electron microscopy and immunofluorescence studies confirmed that autophagic markers were enhanced in the ventral horn of SMA pups. To clarify the role of autophagy, we administered intracerebroventricularly (at P3) either an autophagy inhibitor (3-methyladenine, 3-MA), or an autophagy inducer (rapamycin) in SMA pups. Motor behavior was assessed daily with different tests: tail suspension, righting reflex, and hindlimb suspension tests. 3-MA significantly improved motor *performance*, extended the lifespan, and delayed MN death in lumbar spinal cord (10372.36 ± 2716 MNs) compared to control-group (5148.38 ± 94 MNs). Inhibition of autophagy by 3-MA suppressed autophagosome formation, reduced the apoptotic activation (cleaved caspase-3 and Bcl2) and the appearance of terminal deoxynucleotidyl transferase dUTP nick end labeling (TUNEL)-positive neurons, underlining that apoptosis and autophagy pathways are intricately intertwined. Therefore, autophagy is likely involved in MN death in SMA II, suggesting that it might represent a promising target for delaying the progression of SMA in humans as well.

## Introduction

Spinal muscular atrophy (SMA) is an autosomal recessive neurodegenerative disease selectively affecting motoneurons (MNs) in the spinal cord. SMA symptoms include motor impairment, muscle weakness, and atrophy that lead at the death of infants and toddlers^1^. SMA is caused by inactivation of the evolutionarily highly conserved survival motoneuron 1 (*SMN1*) telomeric gene and subsequent reduction of the full length SMN1 (FL-SMN1) protein, which results in the loss of lower MNs. *SMN1* plays an important role in the maturation of splicing small nuclear ribonucleoproteins^2^. Humans display a second centromeric copy of the gene, *SMN2*, bearing a C to T transition in exon 7. This mutation leads to the lack of exon 7 during transcription and encodes a truncated, less-functional protein, SMNdelta7^3–5^. Wirth et al.^6^ demonstrated that the increase in the copy number of *SMN2* attenuates the symptom severity and delays the age of onset. The low amount of FL-SMN protein in combination with the instability of SMNdelta7 triggers the pathological condition in SMA patients^7^. Despite the genetic cause of SMA is unequivocal, the molecular mechanisms that lead to selective motor neuron (MN) loss and clinical symptoms remain controversial. In order to perform preclinical studies to understand the pathogenesis and to test new therapeutic targets, several in vitro^8^ and in vivo^9^ models have been generated reproducing the hallmarks of the disease. The SMNdelta7 SMA mouse is the most widely used and best-characterized model of SMA (SMN2^+/+^; SMNdelta7^+/+^; Smn^−/−^) with a lifespan of about 13 days^10^. It represents an intermediate SMA form (SMA type II), recapitulating several features of the human disease: behaviorally, for the early motor symptoms and, morphologically, for the degeneration of lower MNs.

Abnormal autophagy has garnered attention as a significant contributor to neurodegeneration^11^. In eukaryotes, autophagy is a physiological process that contributes to various events, such as intracellular cleansing leading to the degradation of long-lived proteins, cytoplasmic organelles, and toxic agents, as by fusion with pre-existing lysosomes^12^. Autophagy consists of several sequential steps: (i) the formation of autophagosomes which are typically labeled by the microtubule-associated protein 1A/1B-light chain 3 (LC3)^13–15^, (ii) fusion with the lysosomes, and (iii) degradation of the cargo by lysosomal hydrolases^16^.

A recent report described the increase of autophagic vesicles in SMN-depleted MN cultures and in MNs cultured from SMA mice, suggesting an increase of autophagosomes without impairment of the autophagic flux^17^. On the contrary, Custer and Androphy^18^ observed an impairment of the autophagic degradative pathway in cell culture and animal models of SMA. Therefore, autophagy dysregulation can represent a new pathogenetic hypothesis in SMA and its pharmacological manipulation could influence the progression of the disease.

Several studies showed that autophagy is intimately intertwined to apoptosis, and same regulators can sometimes control both. In SMAI patients, the relevance of apoptosis has been underscored by several authors. Simic et al.^19^ showed apoptosis is involved in MN degeneration. Soler-Botija et al.^20^ demonstrated a decrease in the levels of Bcl2 and a delay in the expression of Bcl-X in *post-mortem* spinal cords from SMAI foetuses at 15 weeks in comparison with controls. Furthermore, Tsai et al.^21^ found that overexpression of anti-apoptotic Bcl-xL extended the lifespan in a mouse model of SMA. With its dual role in life and death, autophagy assumes a pivotal position in various physiological events such as development and aging, and in many pathological conditions.

Our findings underscore a significant role of autophagy in SMA: its manipulation by 3-methyladenine (3-MA, an autophagic inhibitor) delayed MN degeneration and increased lifespan in the SMNdelta7 mouse model, thus providing a possible therapeutic target in the treatment of SMA.

## Results

### Increased expression of autophagic and apoptotic markers in the lumbar spinal cord of SMA pups

**Fig. 1:**
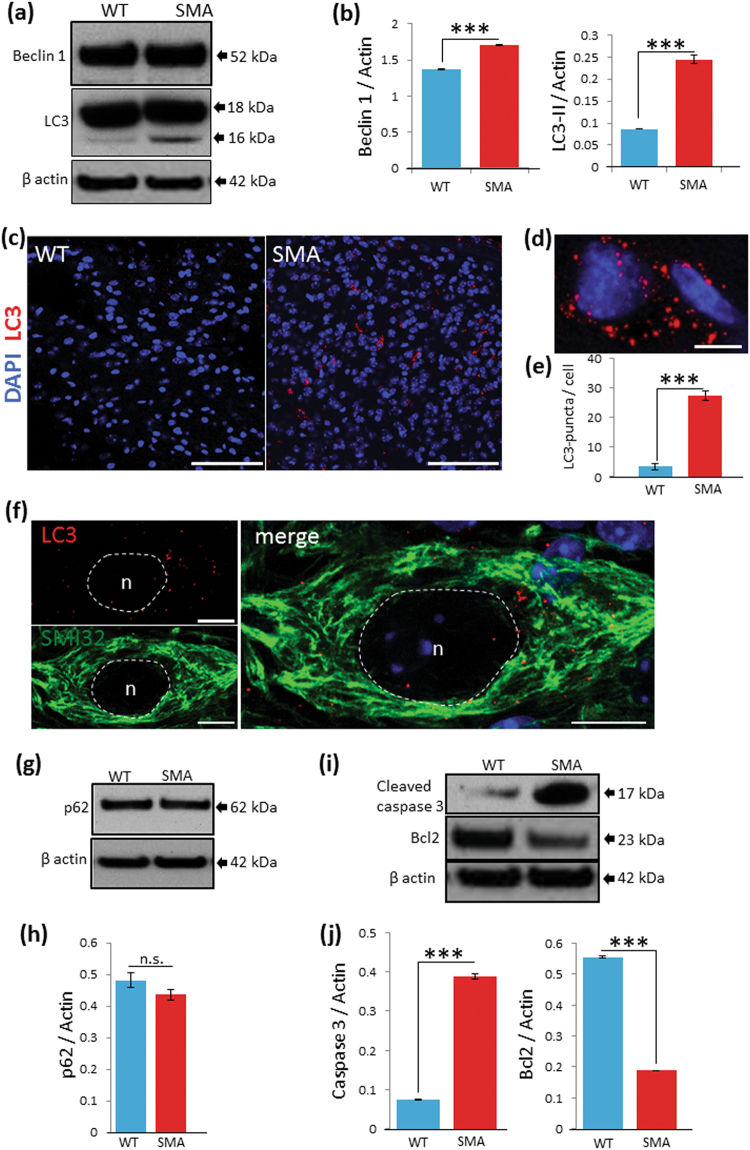
Autophagic markers increase in the spinal cord of SMA pups **a** Representative WB of Beclin 1 and LC3 in the lumbar spinal cord extracts from control (WT) and SMA pups. **b** Beclin 1 and LC3-II levels quantified by densitometry and normalized to β-actin of three independent experiments. Asterisks indicate significant differences using one-way ANOVA test and Bonferroni post-hoc multiple comparisons (***p < 0.001). **c** Immunohistochemical analysis of LC3 (red) in the lumbar spinal cord (ventral horn) at P9 in control (WT) and SMA pups. Nuclei are stained with DAPI (blue). LC3-positive dots are rarely observed in control samples (WT) whereas strong immunoreactivity for LC3 is detected in the spinal cord of SMA pups. **d** High magnification representative image showing increased LC3-positive puncta in the cytoplasm of SMA pups. **e** Graph, showing the corresponding quantification of LC3-positive puncta per cell, indicated as mean value ± SD from WT and SMA pups (***p < 0.001). **f** High magnification representative image of LC3-positive puncta (red) in SMI32-positive MN (green). DAPI (blue) in merge picture. Scale bars: 10 µm. **g**, **h** Protein extracts of lumbar spinal cords in WT (control) and SMA with anti-p62/SQSTM1 antibody by WB analysis. Graph values represent the expression of p62/SQSTM1 normalized with β-actin and correspond to the quantification of three independent experiments. No statistic differences were found between control and SMA groups by one-way analysis of variance (ANOVA), followed by post-hoc Bonferroni test (not significant, n.s.). **i**, **j** Quantitative analysis of repeated WB analysis against cleaved caspase-3 and Bcl2 in SMA in comparison to the WT (***p < 0.001). Scale bars: 100 µm **c** and 5 µm **d**

In order to investigate whether autophagy is specifically implicated in the MN degeneration in SMA mouse model, we analyzed the lumbar spinal cord by western blot (WB) analysis. Beclin 1 protein levels were significantly higher in SMA (1.70 ± 0.004 SD) than in the control (1.37 ± 0.004 SD, ***p < 0.001) (Fig. 1a, b). Moreover, LC3-II protein levels increased significantly in SMA pups (0.24 ± 0.0086 SD) compared to the control (0.09 ± 0.0003 SD, ***p < 0.001) (Fig. 1a, b). Moreover, immunoreactivity for LC3 was strikingly higher in lumbar spinal cord of SMA pups compared to the control (Fig. 1c). High magnification showed a large number of LC3-positive dots in the ventral horns of SMA pups (Fig. 1d). LC3-positive dots counts showed a significant increase in the number of autophagosomes per cell in SMA (27.34 ± 1.64 SD) in comparison to the controls (3.42 ± 0.98 SD, ***p < 0.001) (Fig. 1e). Double-immunostaining showed accumulation of LC3-positive dots in SMI32-labeled MNs in the ventral spinal cords of SMA pups (Fig. 1f).

The increase of autophagosomes could not necessarily indicate an increase in autophagosome biogenesis, but might also be due to a defect in the autophagic flux. In order to discern between these two hypotheses, we measured the protein level of p62/SQSTM1, which is selectively degraded by autophagy^22^. p62/SQSTM1 were unchanged in SMA (0.48 ± 0.024 SD) compared to the control (0.43 ± 0.018 SD, not significant), suggesting that the autophagic flux is unaffected (Fig. 1g, h).

To further investigate the mechanisms of MN cell death in SMA, we assessed the levels of the apoptotic markers, cleaved caspase-3 and Bcl2 in the lumbar spinal cord in SMA and control group. WB analyses showed a remarkable increase in cleaved caspase-3 expression in SMA pups (0.39 ± 0.008 SD) (vs. control 0.075 ± 0.001 SD, ***p < 0.001) (Fig. 1i, j) and Bcl2 levels were reduced in SMA (0.19 ± 0.001 SD) (vs. control 0.55 ± 0.004 SD, ***p < 0.001) (Fig. 1i, j). Therefore, these results suggested also the occurrence of the apoptotic process in SMA.

### Presence of autophagic features in dying lower MNs of SMA pups, assessed by electron microscopy

**Fig. 2:**
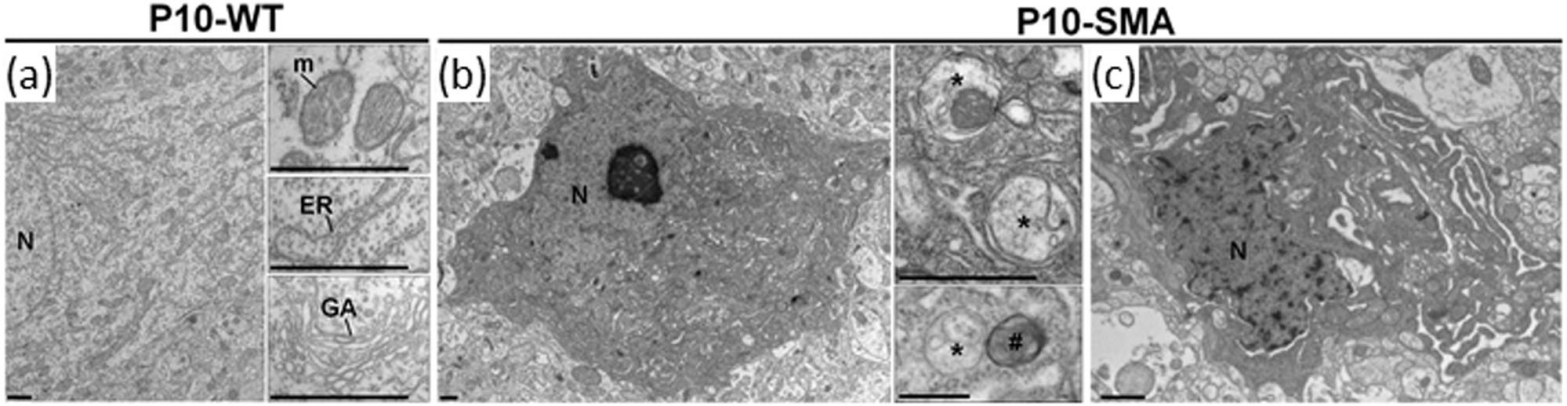
Increased autophagic features in MN in the ventral horn of lumbar spinal cord of SMA pups in comparison to control group **a** Representative electron micrograph of motoneuron cell body in P10 WT mice. High magnifications showed the mitochondria (m), the endoplasmic reticulum (ER) and the Golgi apparatus (GA). **b**, **c** Representative electron micrographs of motoneuron cell body in P10 SMA mice (N: nucleus). High magnifications revealed the presence of both autophagosomes (*) and autolysosomes (#) in the cytoplasm of SMA motoneurons. Dying motoneurons displayed cytoplasmic shrinkage, increased autophagic features (autophagosomes and autolysosomes), swelling of ER and perinuclear membrane and convoluted nuclei with few chromatin condensation (see in **c**). Scale bar: 1 µm

In order to confirm the involvement of autophagy in dying MNs in the SMA model, we performed ultrastructural analysis on the ventral horns of lumbar spinal cords in P5 and P10 mice. Whereas at P5 electron microscopy analysis did not reveal any increase of autophagy and cell death feature in the soma of MNs (not shown), at P10 the cytoplasm of MNs showed marked alterations and signs of cellular disruption. Indeed, most MN cell bodies displayed ultrastructural abnormalities: shrinkage and vacuolization of cytoplasm, swelling of the endoplasmic reticulum and the perinuclear membrane, convoluted nuclei associated with few chromatin condensation and increased autophagic features (autophagosomes and autolysosomes; Fig. 2).

### 3-MA treatment prevents the increase in autophagy occurring in SMA

**Fig. 3:**
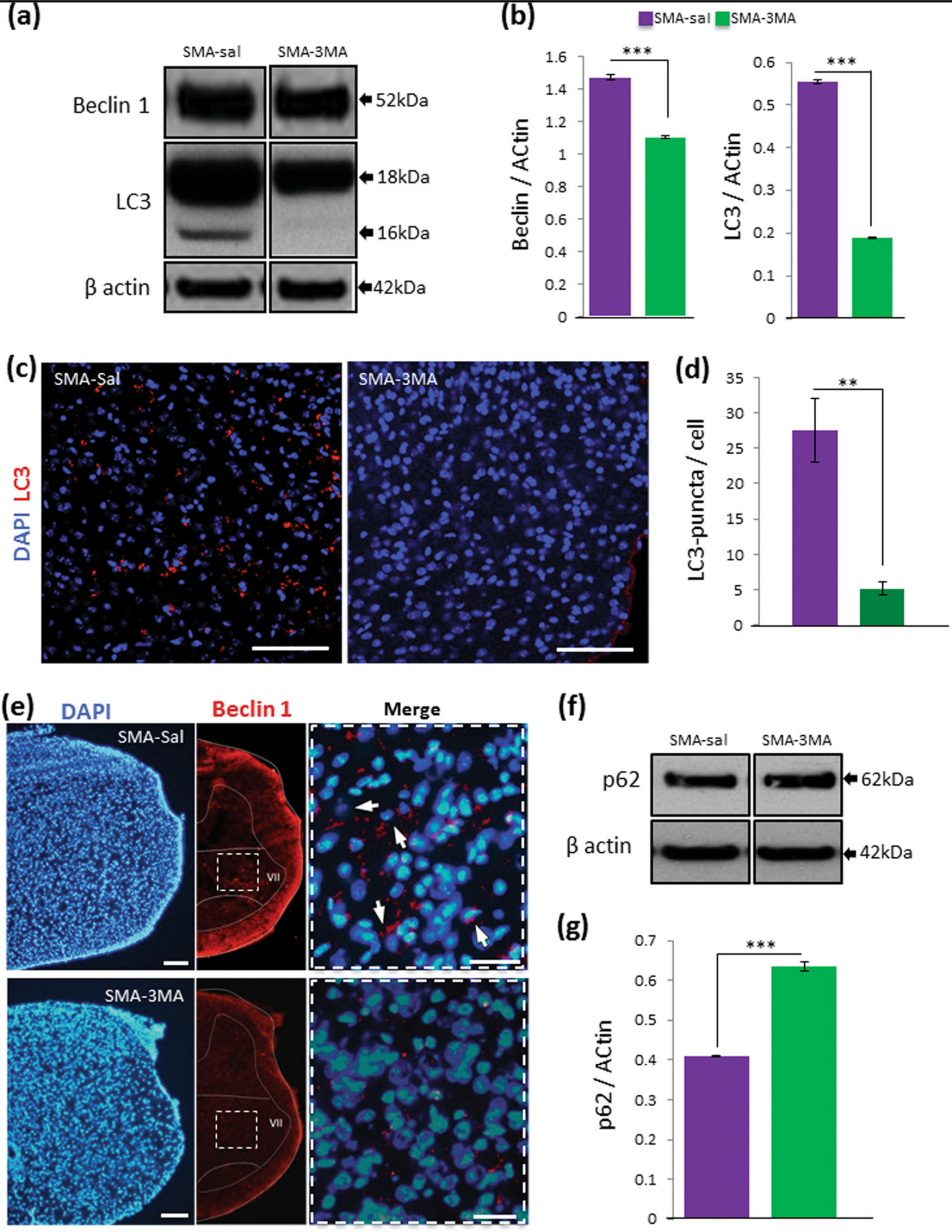
Decrease in autophagic markers and activity in the lumbar spinal cord of SMA after ICV administration of 3-MA **a** Protein extracts of spinal cord of control (WT) and SMA pups with anti-Beclin 1, LC3 and β-actin by WB analysis. **b** Graphs values represent the expression of Beclin 1 and LC3-II normalized with β-actin in SMA pups treated with vehicle (saline) and 3-MA. Results correspond to three independent experiments ±SD. Asterisks indicate significant differences determined by one-way ANOVA, followed by post-hoc Bonferroni test (***p < 0.001). **c** Immunohistochemistry staining using an antibody against LC3 (red) and nuclear staining (DAPI, blue) in SMA treated with vehicle (SMA-saline) and 3-MA (SMA-3MA). Scale bar: 100 µm. **d** Graphs represent the mean of LC3-positive puncta per cell, corresponding to the quantification of three independent experiments ±SD in SMA treated with saline and 3-MA. Asterisk indicates significant difference using one-way ANOVA followed by post-hoc Tukey test (**p < 0.01). **e** Immunostaining of lumbar spinal cord in SMA treated with vehicle (SMA-saline) and 3-MA (SMA-3MA) against Beclin 1 (red) (DAPI, blue). Scale bar: 100 µm (20 µm, high magnification). **f** WB analysis of p62/SQSTM1 in SMA treated with saline or 3-MA. **g** Protein levels of p62/SQSTM1 by densitometry and normalized to β-actin. Results are shown as the mean value ± SD (mean of three independent experiments). Asterisks indicate significant differences by one-way ANOVA test, followed by post-hoc Bonferroni test (***p < 0.001)

To further explore the role of autophagy in the pathogenesis of SMA disease, we performed the intracerebroventricular (ICV) administration of 3-MA, a common autophagy inhibitor, at P3 (SMA-3MA group), and we evaluated if the modulation of autophagy could influence the disease progression. Quantification analyses indicated that Beclin 1 was reduced in SMA after 3-MA administration in comparison to the control (SMA-saline, 1.47 ± 0.02 SD; SMA-3MA, 1.10 ± 0.004 SD, ***p < 0.001) (Fig. 3a, b). Also LC3-II protein levels were significantly decreased in SMA pups after 3-MA administration (SMA-saline, 0.61 ± 0.008 SD; SMA-3MA, 0.18 ± 0.009 SD, ***p < 0.001) (Fig. 3a, b). Immunostaining against LC3 showed a relevant reduction in SMA-3MA group compared with SMA-saline pups (Fig. 3c). LC3-positive puncta counts showed a significant reduction in the number of autophagic vesicles per cell after 3-MA treatment (5.15 ± 0.93 SD) in comparison to SMA-saline group (27.5 ± 4.51 SD) (Fig. 3d, **p < 0.01). Moreover, Beclin 1 immunoreactivity was strongly reduced after 3-MA treatment in comparison with vehicle group (Fig. 3e). After 3-MA administration, p62/SQSTM1 protein level was significantly increased (SMA-3MA 0.64 ± 0.026 SD; ***p < 0.001) in comparison to the control groups (SMA-saline 0.40 ± 0.06 SD), suggesting a reduction of autophagy activity (Fig. 3f, g). Altogether, these results suggested an inhibition of the autophagy pathway in the lumbar spinal cord after the ICV administration of 3-MA.

To determine whether changes in SMN after 3-MA treatment can be detected in isolated MNs from an in vivo SMA model, we used the Smn^−/−^;SMN2^+/+^ (mutSMA) mouse. We dissected and genotyped E13 embryos by crossing two Smn^+/−^;SMN2^+/+^ mutants. LC3-II protein was increased in mutSMA cultures compared with WT control cells (1.69 ± 0.12, *p* < 0.05, data not shown). However, LC3-II levels were reduced in WT and mutSMA 3-MA-treated cells (0.69 ± 0.08, *p < 0.05 and 0.65 ± 0.04, ***p < 0.001, respectively) compared with non-treated conditions (SF. 1a). Additionally, Smn protein level was increased in both WT and mutSMA 3-MA-treated conditions (1.34 ± 0.12- and 1.74 ± 0.31-fold increase, *p < 0.05, respectively) compared with non-treated controls (SF. 1b). We measured p62/SQSTM1 in WT and mutSMA conditions and we observed significant increase in mutSMA (2.12 ± 0.31-fold increase, *p* < 0.05, data not shown) compared with WT control. 3-MA treatment increased p62/SQSTM1 in WT-treated cells (1.35 ± 0.08-fold induction, **p < 0.005) compared with non-treated cells, but no significant differences in p62/SQSTM1 level were observed in 3-MA-treated and non-treated mutSMA conditions (SF. 1c).

### Autophagy manipulation modulates MN degeneration in SMA mice

**Fig. 4:**
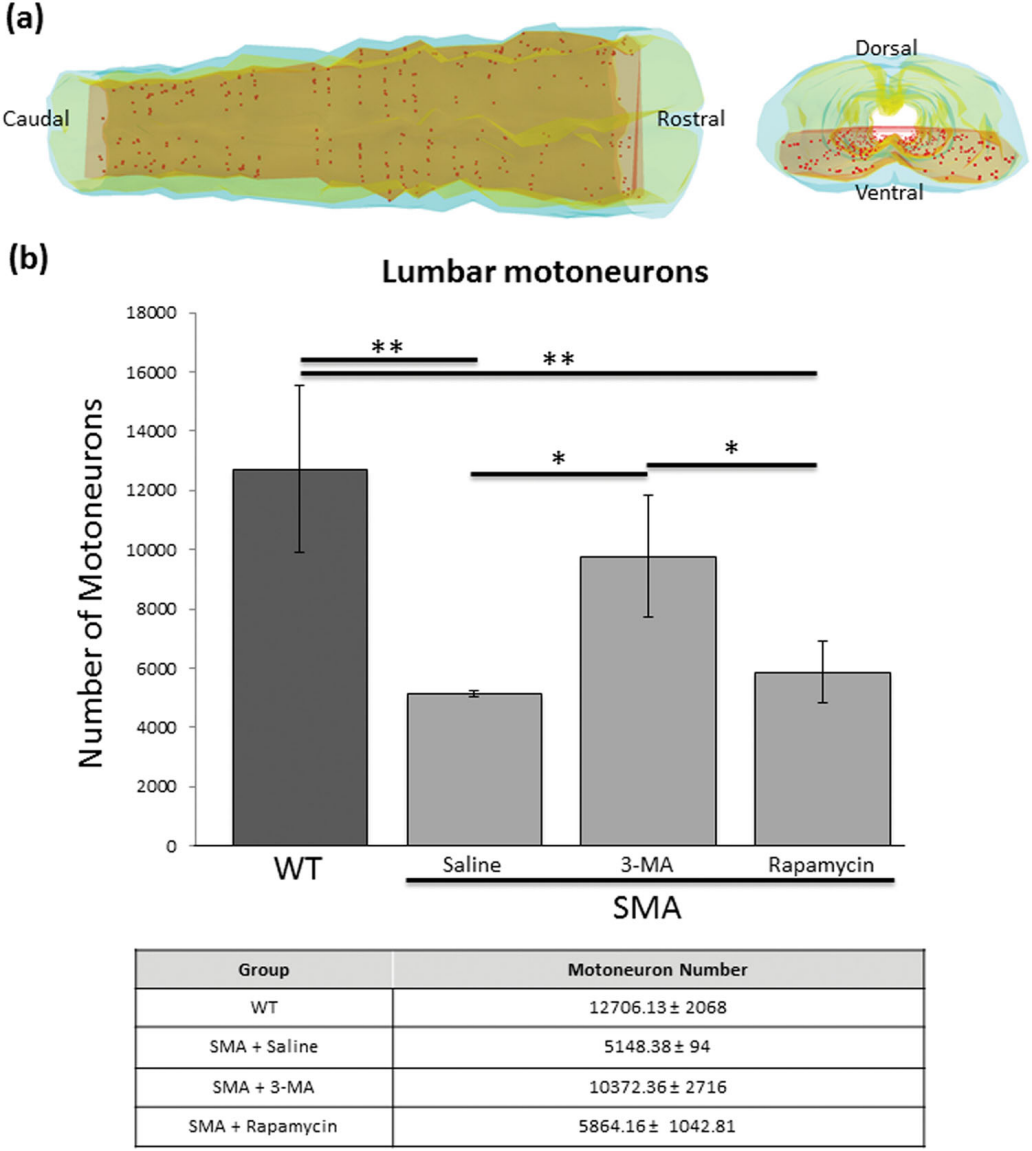
Effects of autophagy inhibition on MN survival **a** Example of an SMA P9 lumbar spinal cord three-dimensional reconstruction by StereoInvestigator software. Schematic dorsal view (left) and transverse section (right) of the lumbar spinal cord show the localization of the MNs (red dots) in the ventral part (red volume). The white matter is in cyan and gray matter in yellow. **b** Graph and table indicate the number of MNs (stereological counts) in the lumbar spinal cord (ventral horn) at P9 in WT, SMA-saline, SMA-3MA, and SMA-Rapamycin (SMA-RAP) groups. Statistical difference between the groups are indicated (*p < 0.05; **p < 0.01)

To evaluate how autophagy manipulation can influence MN death, we performed stereological counts of MNs in Nissl-stained sections (Fig. 4). The analyses were performed on lumbar spinal cords of P9 WT and SMA pups treated with vehicle (SMA-saline) and 3-MA (SMA-3MA) or rapamycin (SMA-RAP). The number of MNs was greatly decreased in SMA-saline (5148.38 ± 94 SD MNs/lumbar spinal cord) and SMA-RAP groups (5864.16 ± 1042.81 SD) compared to WT (12706.13 ± 2068 SD, respectively *p* = 0.002 and *p* = 0.005, **); however, the SMA-3MA group showed a significantly increased number of MNs (10372.36 ± 2716 SD) when compared to SMA-saline and SMA-RAP (respectively *p* = 0.02 and *p* < 0.05, *), and a not significant difference compared to WT (Fig. 4b). These results suggest that 3-MA treatment delays MN degeneration in the lumbar spinal cord of SMA pups.

### Relationship between autophagy and apoptosis

**Fig. 5:**
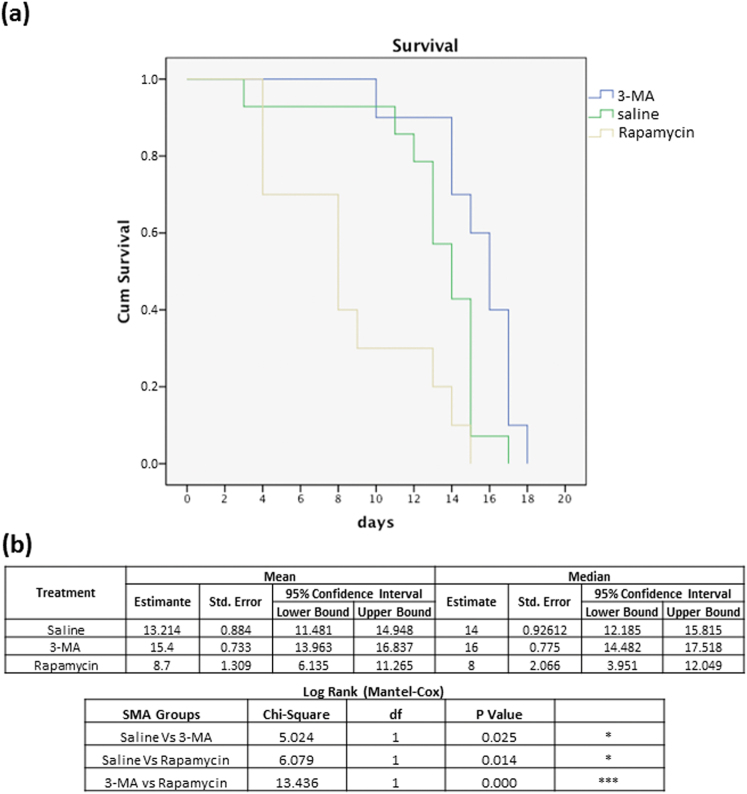
Administration of 3-MA reduces apoptotic cell death in the lumbar spinal cord of SMA **a** Protein extraction of lumbar spinal cord from different groups. **b** Quantitative analysis of repeated WB analysis against cleaved caspase-3 and Bcl2. After 3-MA administration, results indicate reduction of cleaved caspase-3 protein level in SMA in comparison to SMA-saline group (**a**, **b**). Quantitative analysis of repeated WB shows increased level of Bcl2 after 3-MA administration (**a**, **b**). **c** Staining shows the presence of cleaved caspase-3-positive cells (**c** and high magnification **c'**, red) and TUNEL-positive cells (**d** and high magnification **d** **'**, green) in the ventral horn in the lumbar spinal cord of SMA-saline but absence of positive cells in SMA-3MA group. Nuclear staining (DAPI, blue). Scale bar: 50  m (5 μm, insert)

We analyzed the protein levels of cleaved caspase-3 and Bcl2 in the lumbar spinal cord after 3-MA administration. WB analyses showed a remarkable reduction of cleaved caspase-3 in SMA-3MA mice (0.25 ± 0.0044 SD, ***p < 0.001) in comparison to SMA-saline (0.34 ± 0.008) (Fig. 5a, b). In addition, Bcl2 levels were increased (SMA-3MA, 0.77 ± 0.008 SD, ***p < 0.001) compared to the SMA-saline group (0.48 ± 0.002 SD) (Fig. 5a, b). Immunohistochemical analysis showed that cleaved caspase-3 was absent in SMA pups after 3-MA treatment, whereas some positive cells could be detected in SMA-saline (Fig. 5c and insert 5c'). Terminal deoxynucleotidyl transferase dUTP nick end labeling (TUNEL)-staining gave similar results (Fig. 5d and insert 5d'). Taken together, our results showed that the 3-MA administration reduced cell death, underlying the relationship between autophagy and apoptosis in the control of MN degeneration in the spinal cord of SMA mice.

### Modulation of autophagy affects the survival of SMA mice

**Fig. 6:**
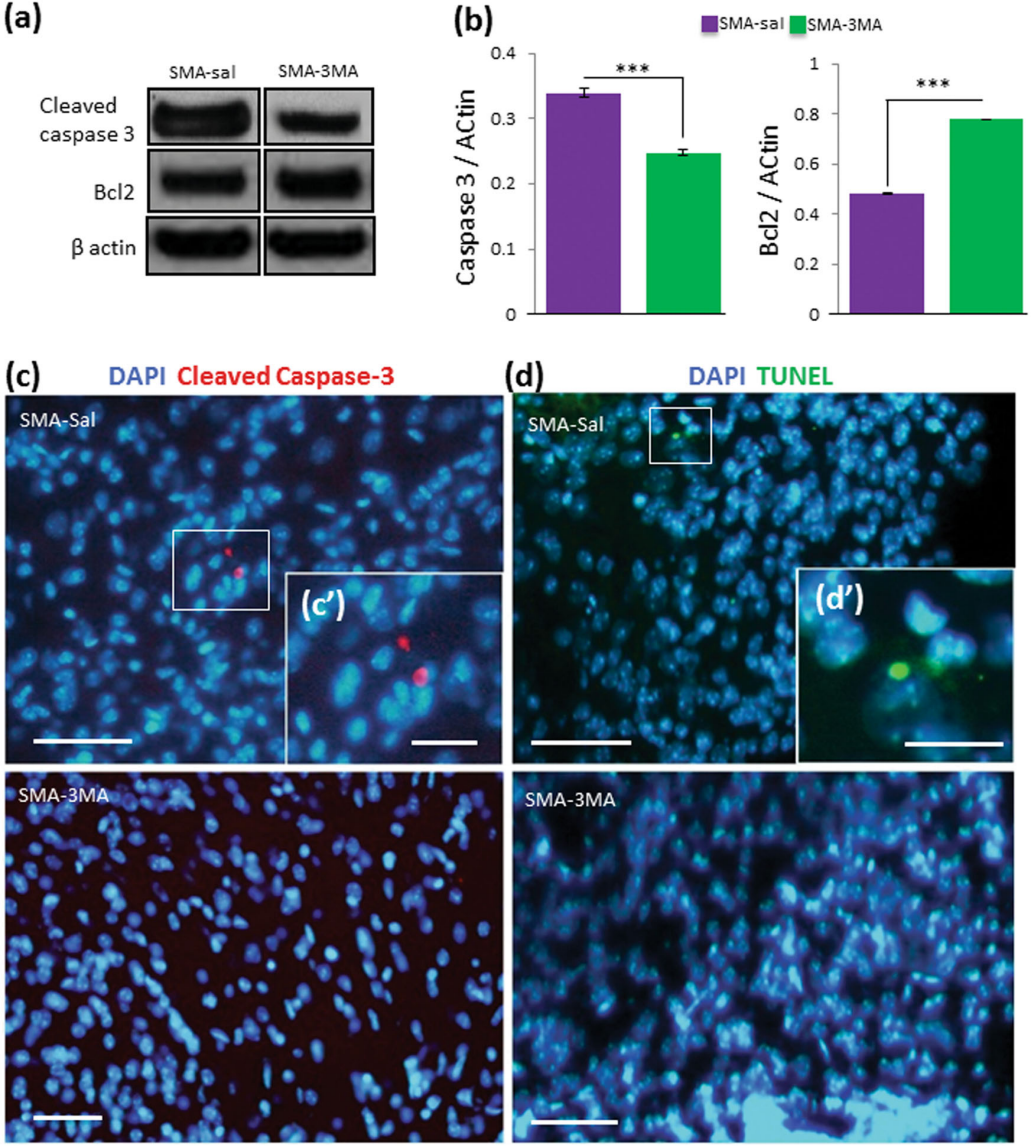
Effect of 3-MA on the lifespan in the SMA pups **a** The results of Kaplan−Meier survival analysis indicate the probability of survival in the vehicle-, 3-MA- and rapamycin-treated SMA pups. **b** The significant difference between the groups

We found that 3-MA administration slightly, but significantly, increased the lifespan of SMA pups (*N* = 10, 15.4 vs. 13.21 days; *p* = 0.025; Fig. 6). On the contrary, SMA-RAP (*N* = 10) pups showed a significant reduction of lifespan compared with SMA-saline (*N* = 14, *p = 0.014) and SMA-3MA (***p = 0.001).

### Autophagy involvement in disease progression in SMA mice

**Fig. 7:**
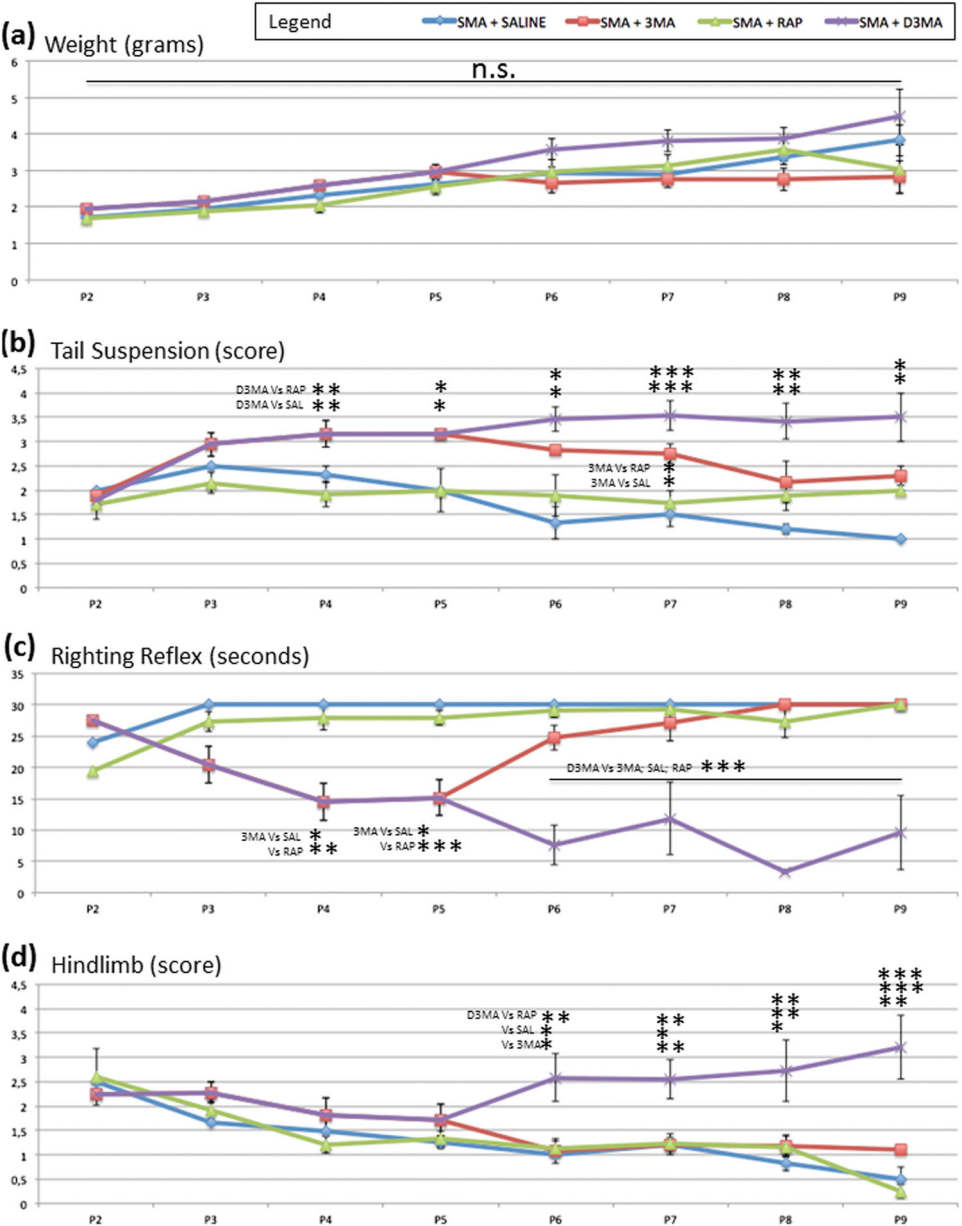
Body weight and behavioral tests in SMA pups Analysis of body weight and motor behavior (from P2 to P9) of SMA treated with saline (blue line), rapamycin (green) and single (P3, red) or double (P3 and P6, purple) administration of 3-MA. The grams, score, and the seconds are expressed as mean±SD. Statistical difference between the groups are indicated (*p < 0.05; **p < 0.01; ***p < 0.001) (not significant, n.s.)

In order to evaluate the effects of modulating autophagy in the progression of disease, body weight, and a battery of behavioral tests were performed daily. All mice were observed from P2 to the killing (P9), and subdivided in four SMA groups: saline-treated (SMA-SAL, *N* = 3), 3-MA-treated (SMA-3MA, *N* = 5–14), rapamycin-treated (SMA-RAP, *N* = 3–13), and D3-MA-treated (double administration of 3-MA at P3 and P6, *N* = 3–6) (SMA-D3MA) (Fig. 7).

#### Body weight

No significant differences were found among the four groups (Fig. 7a).

#### Tail suspension test

This test measures motor coordination in pups. The performance before the treatment was overlapping among the groups. After 3-MA treatment, pups showed a better performance in comparison to the SMA-SAL and SMA-RAP groups, but the positive results were transient. Therefore, we created a group of SMA pups with a double administration of 3-MA (P3 and P6), which showed a strong improvement in motor behavioral test to P3 from P9 (Fig. 7b).

#### Righting reflex test

This simple assay was performed to evaluate motor coordination. The SMA-SAL and SMA-RAP pups failed the test. When 3-MA was administrated at P3, pups recovered their righting reflex. At P4 and P5, SMA-3MA pups had improved (P4: 14.53 ± 2.99 SEM; P5: 15.18 ± 2.84 SEM) in comparison to SMA-SAL (P4: 30, *p = 0.033; P5: 30, *p = 0.012) and SMA-RAP (P4: 28 ± 2 SEM, **p = 0.003; P5: 27.92 ± 1.15 SEM, ***p = 0.001). However, 3 days after the administration (P6), the SMA-3MA (24.75 ± 1.97 SEM) pups progressively lost their capability and performed similarly to SMA-SAL (30) and SMA-RAP pups (29 ± 1 SEM). These results showed a transient positive effect of 3-MA. SMA pups following double administration of 3-MA, the improvement was consistent and lasted from P3 to P9 (P6: 7.6 ± 3.19 SEM; P7: 11.87 ± 5.77 SEM; P8 = 3.33 ± 0.33 SEM; P9: 9.57 ± 5.95 SEM, ***p < 0.001) (Fig. 7c).

#### Hindlimb suspension test

This test evaluates hindlimb strength and coordination. SMA-D3MA pups improved motor performance, in absence of differences among other groups (Fig. 7d).

All these data suggest that autophagy inhibition might reduce the disease progression in SMA model.

## Discussion

Autophagy is an evolutionarily conserved mechanism across eukaryotes which removes damaged organelles and protein aggregates from the cytoplasm. The essential steps of autophagy involve several autophagy-related (ATG) proteins^15,23^. Throughout their life, neurons are particularly dependent on autophagy to maintain their homeostasis and neuron-specific knockout for Atg5 or Atg7 showed axonal degeneration and neuron death in mice^24–27^. However, autophagy is considered a Janus-faced mechanism, which can alternatively either protect from or contribute to neuronal damage: for example, the accumulation of autophagic vesicles has been reported in human post-mortem brains of patients affected by neurodegenerative disorders^28,29^ and in mouse models of neurodegeneration^30–32^. Autophagy has been suggested as a fundamental process whose dysregulation can be also involved in neurodegenerative diseases and become, finally, a potential target for therapy. A dysregulation of autophagy has been reported in SMA: Garcera et al.^17^ provided the first evidence, reporting an increased number of autophagic features in cultured CD1 mouse model after lentiviral short hairpin RNA Smn-reduction. Periyakaruppiah^33^ revealed an increase of autophagic vesicles in Smn-reduced spinal cord MNs from a murine model of severe SMA, suggesting that autophagy is dysregulated. Moreover, Custer and Androphy^18^ observed the presence of autophagic vesicles in an inducible NSC-34 cell culture model of SMA, in fibroblasts isolated from SMA patients and in spinal cord extracts of “Taiwanese” mouse model of SMA. According to these results, a recent report described an increase of mRNA expression level of autophagy markers in the spinal cord of a severe mouse model of SMA^34^.

Our results in the delta7 in vivo model are in agreement with these previous reports. We observed increased Beclin 1 and LC3-II protein levels in the lumbar spinal cord of the SMA mouse model. In addition, immunofluorescence staining and electron microscopy results showed an increased number of autophagic vesicles in the lumbar MNs in SMA mice.

Autophagosomes might accumulate when autophagosomal digestion is impaired. Therefore, we checked the level of p62/SQSTM1, a marker of autophagic flux^22^. Previous in vitro studies using SMN-depleted cells^18,33^ demonstrated increased p62/SQSTM1 protein levels suggesting a decreased autophagic flux. Custer and Androphy^18^ reported similar results in a severe mouse model of SMA. On the contrary, in our intermediate mouse model of SMA, the p62/SQSTM1 protein levels were unchanged in the spinal cord compared to the control group. Therefore, in our mouse model, autophagy was upregulated, but the autophagic flux was unaffected. In support of this hypothesis, Garcera et al. suggested a normal autophagic flux in an in vitro model of SMA^17^ showing increased LC3-II level after Bafilomycin treatment.

Several studies suggested that autophagosome accumulation may interfere with intracellular trafficking and/or may become a source of cytotoxic products^35,36^. In order to understand whether reduction of autophagosomes could improve the pathogenesis of the disease, we treated the SMA pups with 3-MA, an autophagy inhibitor. The levels of Beclin 1 and LC3-II decreased in the lumbar spinal cord of SMA mice following ICV administration of 3-MA and the number of LC3-positive vesicles reduced in 3-MA-treated SMA pups. In accordance with these data, the p62/SQSTM1 protein levels increased after 3-MA administration. These data demonstrated that 3-MA suppressed autophagosome formation and autophagy activity in the lumbar spinal cords of SMA mouse model. Then, we performed stereological MN counts in the lumbar spinal cord and we measured the lifespan and motor function of the animals. Our study showed that 3-MA delayed MN degeneration and improved motor behavior temporarily and slightly but significantly increased the lifespan, confirming the relevance of autophagy in SMA neurodegeneration and suggesting for the first time that modulation of autophagy by 3-MA may affect disease progression. The relevance of modulating autophagy through 3-MA is further highlighted by the results we obtained in vitro, where it increased SMN protein levels both in WT and in mutSMA cultured MNs. This corroborates the hypothesis of a strong relationship between autophagy and SMN, completely aimed at supporting/maintaining the healthy MN state. In support of our observation, Olivan et al.^34^ also described the neuroprotective effects, due by the reduction of autophagy markers and pro-apoptotic gene using a non-viral gene therapy in a mouse model of SMA.

Furthermore, we investigated the link between autophagy and apoptosis. According to previous studies in the spinal cord of children with the severe form of SMA^19^ and in mouse models of SMA^20,21^, we observed increased cleaved caspase-3 and reduced Bcl2 protein levels compared to the control. Interestingly, 3-MA treatment reduced apoptotic cell death in the lumbar spinal cord, underlining the important relationship between autophagy and apoptosis, as already suggested by several authors^32,37–39^. Inhibitors of autophagy can trigger^40^ or inactivate apoptosis^41^ depending on the context. Several authors demonstrated that Bcl2 family members inhibit autophagy by linking Beclin 1^37^. Altogether, these results suggest that Beclin 1-dependent autophagy is modulated in our experimental SMA model and could determine MN degeneration.

However, autophagy in MN diseases plays a controversial role, as reviewed recently^42^. For example, in amyotrophic lateral sclerosis (ALS), the survival of MNs is affected by impaired autophagy^43^. Impairment of the retrograde axonal transport in SOD1 G93A mice may be responsible for the accumulation of autophagosomes as a result of a defect in the autophagosome−lysosome fusion, which occurs mainly in the soma^44^. Despite several lines of evidence highlighting the role of autophagy in ALS pathogenesis^45^, knocking-out MN autophagy proved to be insufficient to replicate ALS in mice^46^. Nevertheless, it has been shown that in ALS aggregated misfolded proteins, such as TDP-43 and SOD1, may form and accumulate in MN soma and axons^47,48^, thus activating autophagy^49^. The inhibition of autophagy by 3-MA may induce aggregates containing TDP-43^50^. On the contrary, the inhibition of mTOR using rapamycin results in the induction of autophagy, relocalization of TDP-43 to its proper nuclear compartment and reduction of its accumulation, and rescue of mRNA processing^51^. In addition, enhancing autophagic flux through an mTOR-independent pathway with trehalose inhibited TDP-43 aggregate formation^52^ and autophagy-activating molecules increased TDP-43 clearance and improved neuronal survival in ALS models^53^. The positive effect of upregulating autophagy in ALS has been pointed out in SOD1 models^54,55^. This evidence was also supported by work using *litium* salts as autophagy inducers, having a neuroprotective effect^56^. Also, the same authors have shown that, in glutamate-induced toxicity in MNs, cell death is associated to impaired autophagy^57^. Conversely, Zhang et al.^58^ found that rapamycin treatment enhances MN degeneration in a mouse model of ALS. Therefore, to summarize findings on autophagy and ALS, it seems that autophagy plays a protective role, even though its induction is not sufficient to rescue MNs, and several controversial observations are still reported.

Even though ALS and SMA are both MN diseases, and share some histopathological findings, their etiopathogenesis, albeit multifarious, is fundamentally different for the genes involved and the age of disease onset and progression.

The effects of autophagy manipulation in SMA are in partial disagreement to what occurs in ALS, since 3-MA by blocking autophagy protects MNs. Consequently, our findings suggested that drugs that induce autophagosome formation should not be considered for the treatment of SMA.

Moreover, the repeated administration of 3-MA showed improved motor performance in SMA pups, thus suggesting that this strategy should be considered for further studies and analysis. However, chronic use of 3-MA may have some limitations. This inhibitor reduces autophagy by its action on class III phosphatidylinositol 3-kinase (PtdIns3K), but it also affects the class I, which activates Akt pathway^59^. Therefore, even though 3-MA represents an effective inhibitor of autophagy, its therapeutic potential is limited by virtue of its action on class I PtdIns3k because, on the contrary, it can equally promote autophagy especially after long exposure^59^. Thus, it will be of interest to investigate the effects of other autophagy inhibitors, e.g. LYR294002 and Wortmannin (reviewed in ref.^60^).

In summary, in SMA autophagy seems to act more as a death mechanism (in association with apoptosis, as we showed) than being neuroprotective. On the other hand, the distinction between the two roles could be not so rigid, and the final effect of autophagy could be more related to its fine tuning than to an all-or-none effect.

To this extent, the tight regulation of autophagy could be a good strategy to increase the therapeutic potential as a complementary therapy against SMA, possibly in combination with the emerging promising treatments aimed at manipulating the SMN expression (e.g. nusinersen)^61,62^.

## Materials and methods

### In vivo experiments

#### Animals

Experiments were performed on newborn SMNdelta7 mice (Jackson Laboratories; stock number 005025) from the animal colony bred in the Neuroscience Institute Cavalieri Ottolenghi at the University of Turin. Mice were housed with 12-h light/dark cycle and were given free access to food and water ad libitum. All animal experimental procedures were in accordance with the European Communities Council Directive of 24 November 1986 (86/609/EEC), the National Institutes of Health guidelines, the Italian law for care and use of experimental animals (DL116/92), and approved by the Italian Ministry of Health and the Bioethical Committee of the University of Turin (authorization number 17/2010-B, 30 June 2010).

#### Genotyping

The mice were genotyped using DNA isolated from a small piece of tail. Isolation was performed with proteinase k (50 µg) in lysis buffer (10 mm Tris HCl, 50 mm KCl, 0.01% gelatin, 0.45% IGEPAL, 0.4% Tween-20) at 55 °C overnight under gently shaking. The presence of the transgene was determined by PCR analysis using primers that amplify a portion of the *smn* gene, yielding a 420 bp product for the wild-type allele and a 150 bp product for the transgenic one. They were: smn fwd 5′-TTTTCTCCCTCTTCAGAGTGAT-3′, smn wt rev 5′-CTGTTTCAAGGGAGTTGTGGC-3′ and smn tg rev 5′-GGTAACGCCAGGGTTTTCC-3′ as suggested by suppliers (Jackson Laboratories).

#### Phenotyping

In order to analyze the progression of motor symptoms in SMA mice model^63^, we daily checked the body weight and performed behavioral tests, starting at P2.

(i) *Tail suspension test (self-clasping)*: The pups were suspended by the tail (15 s) and we evaluated the hindlimb posture. The score was: 4, normal, hindlimbs spread open; 3, not completely spread; 2, often close; 1, always close together; 0, always close together and in additional postural abnormalities were present (clasping).
(ii) *Righting reflex*: We placed the pups on their backs on a flat surface and we evaluated their ability to right themselves within 30 s.
(iii) *Hindlimb suspension test*: The test, developed at PsychoGenics Inc, evaluated the proximal hindlimb fatigue, weakness, and muscle strength in pups. Each animal was given three trials and the average was recorded. Mice were hanged by their hindlimbs from the border of standard 50 ml plastic centrifuge tube filled with a cotton ball cushion at the bottom. We evaluated the posture (score). The score was divided into four criteria: 4, when the pup has a normal hindlimb separation with tail raised; 3, hindlimbs were closer together but they rarely touch each other; 2, hindlimbs were close to each other and usually touching; 1, weakness apparent and the hindlimbs were almost always clasping; 0, hindlimbs always were in clasping.

### Stereotaxic surgery and administration of chemical modulators of autophagy

To manipulate the autophagy pathway, we performed ICV administration of an autophagy inhibitor, 3-MA (9.8 µg/2 µl, Sigma-Aldrich)^64,65^ or an inducer, rapamycin (2 ng/2 µL)^64,66^, in P3 SMA pups. A sham-operated group was injected with 2 µl of sterile saline (vehicle). Furthermore, another group received 3-MA twice, at P3 and at P6 (D3MA-treated group).

Briefly, neonatal mice were anesthetized by hypothermia (total duration 3–5 min) and their heads were immobilized on a custom neonatal stereotaxic apparatus at 4 °C during surgery. 3-MA was injected at stereotaxic coordinates of 0 mm from bregma, 0.8 mm lateral to sagittal sinus, and 1.5 mm deep^67^ with a Hamilton microsyringe. The pups were then placed on a heat pad, quickly revitalized and subsequently returned to their mother.

### Tissue processing and immunohistochemical procedures

At postnatal day 9 (P9), the pups were deeply anesthetized and perfused transcardially with 4% paraformaldehyde in 0.1 m phosphate buffer (PB), pH 7.4. The lumbar spinal cord was dissected out, post-fixed in the same fixative for 3 h, cryoprotected overnight in 30% sucrose in 0.1 mPB (pH 7.2), embedded in cryostat medium (Killik; Bio-Optica) and frozen. Sections were cut on the cryostat at a thickness of 10 µm, mounted onto gelatin-coated Superfrost® slides, and stored at −20 °C until they were reacted for immunohistochemistry. For immunofluorescence, the sections were washed in phosphate-buffered saline (PBS). After immersion in blocking solution for unspecific binding sites 30 min at room temperature (RT) with 0.3% Triton X-100 and 15% normal donkey serum (NDS, Sigma-Aldrich) in PBS pH 7.4, the sections were incubated overnight at 4 °C with the following antibodies:

- rabbit polyclonal anti-LC3 antibody (anti–Microtubule associated proteins 1A/1B light chain 3; 1:200, Sigma-Aldrich: catalog # SAB4300571, Lot# 871521402, a synthetic peptide made around aa. 5–10, according to the protein); LC3 is the only identified mammalian protein that stably associates with the autophagosome membranes. Immunoblots visualized double bands at ~18 kDa (LC3-I) and ~16 kDa (LC3-II), respectively, characteristic of this protein.
- rabbit poyclonal anti-Beclin 1 antibody (1:200, AbCAM: catalog # ab16998). Beclin 1 plays a key role in the recruitment of other AuTophaGy-related proteins (ATG) involved in the expansion step. In addition, Beclin 1 can interact with Bcl-2 and Bcl-xL to regulate both autophagy and apoptosis.
- mouse monoclonal anti-SMI32 antibody (1:1000, Covance: catalog # SMI-32R). SMI32 visualizes neuronal cell bodies, dendrites and some thick axons in the central and peripheral nervous system; it is one of the most used markers for MN labeling.
- rabbit poyclonal anti-cleaved-caspase-3 antibody (1:400, Cell Signaling Technology: catalog # 9661, detects endogenous levels of the large fragment, i.e. 17/19 kDa, of activated caspase-3). Caspase-3 is a critical executioner of apoptosis, as it is either partially or completely responsible for the proteolytic cleavage of many key proteins.

The antibodies were diluted in PBS containing 1.5% NDS and 0.3% Triton X-100. After rinsing, primary antibodies were detected by incubating sections for 1 h at room temperature in 1:100 DyLight 488-conjugated donkey anti-rabbit or anti-mouse IgG (H+L) and 1:200 cyanine 3-conjugated donkey anti-rabbit or anti-mouse IgG (H+L) (Jackson Immuno Research Laboratories). The sections were counterstained with 4′,6′-diamidino-2-phenylindole (DAPI, Sigma-Aldrich, catalog # d9564), rinsed, coverslipped in 1:1 PB-glycerol, and observed with a Nikon Eclipse E90i epifluorescence microscope under appropriate filters and photographed by a Nikon DS-5Mc digital camera. To create 3D reconstructions, some preparations were also observed with a Leica TCS SP5 confocal laser scanning microscope (Leica, Mannheim). To verify the specificity of the secondary antibodies control sections was reacted similarly, except the primary antibody was omitted in incubation. No immunolabeling was seen in control sections (data not shown).

### Nissl staining and stereological cell counts

The lumbar spinal cord (P9) was serially cut in 20 μm-thick sections and every fifth sections were evaluated (100 μm intervals). Section were mounted onto 2% gelatin-coated Superfrost^®^ slides and air-dried. Slides were hydrated in distilled water for 1 min and stained with 0.1% cresyl violet solution for approximately 8 min. The sections were immersed in ascending alcohols, cleared in xylene and cover-slipped with Eukitt (Bioptica). After Nissl staining, the nucleoli of the lower MNs in the ventral horns of the lumbar spinal cord were stereologically counted at 40×. Only multipolar neurons located in the ventral somatic columns were counted. A total number of MNs were obtained with the Optical Fractionator^68^ by using a computer-assisted microscope and the StereoInvestigator software (MicroBrightField). MNs were counted on the computer screen using an Optronics MicroFire digital camera mounted on a Nikon Eclipse E600 microscope. Ventral gray matter volume of the reconstructed lumbar spinal cord segments was obtained using NeuroExplorer software (MicroBrightField). Every fifth 20 μm-thick Nissl-stained section was reconstructed at P9. The counting frame size was 75×75 μm and the sampling grid size 100×100 μm.

### TUNEL staining

Cell apoptosis was assessed in the lumbar spinal cord of pups at P9 by The DeadEnd™ Fluorometric TUNEL System (Promega, Madison) following the manufacturer’s instructions. DAPI was employed to stain nuclei.

### Electron microscopy

A separate set of animals was killed for electron microscopy analysis (P5 WT *n* = 3, P5 SMA *n* = 3, P10 WT *n* = 3, P10 SMA *n* = 3). Pups were anesthetized with gaseous anesthesia (3% isoflurane vaporized in O_2_/N_2_O 50:50) and perfused intracardially with saline solution pH 7.4 followed by fixative (2.5% glutaraldehyde and 2% paraformaldehyde in PB 0.1 m, pH 7.4). The lumbar spinal cord (L1−L5) was rapidly dissected and post-fixed overnight at 4 °C in the same fixative. Samples were then processed for electron microscopy as previously described^69^. Briefly, samples were postfixed in osmium tetroxide (1% in PB 0.1 m) for 1 h and contrasted in uranyl acetate (1% in 70% ethanol) for 20 min. Spinal cords were then dehydrated in graded alcohols and embedded in Durcarpan ACM resin (Fluka, Neu-Ulm). Regions of ventral horns were identified and ultrathin sections (from silver to gold) were cut with an ultramicrotome and a diamond knife (Diatome). Sections were mounted on formvar-coated single slot grids. Sections were finally visualized using a Philips CM100 transmission electron microscope.

### Immunoblotting

Animals were killed at P9, spinal cords were dissected and immediately homogenized in a glass-Teflon Potter homogenizer in an ice-cold lysis buffer containing 20 mm Hepes, pH 7.5/10 mm KCl/1.5 mm MgCl_2_/1 mm ethylenediaminetetraacetic acid/1 mm ethylene glycol tetraacetic acid/1 mm DTT/ 0.5% 3-[(3-cholamidopropyl)dimethylammonio]-1-propanesulfonate (CHAPS)/complete protease inhibitors; Roche Cat. No. 11 697 498 001); the homogenates were centrifuged at 12,000 rpm for 15 min at 4 °C. Protein concentration was determined using a Bradford assay (#23236). Proteins extracts (50 µg) were separated on sodium dodecyl sulfate polyacrylamide gel electrophoresis (SDS-PAGE) (12% polyacrilamide) and transferred to polyvinylidene difluoride membranes. The membranes were blocked in 5% nonfat milk in tris-buffered saline (TBS)-T (200 mm Tris and 1.5 m NaCl with 0.1% Tween 20) and were incubated with primary antibody diluted in TBS-T overnight at 4 °C.

The membranes were washed and incubated with secondary horseradish peroxidase-coupled antibodies (Bio-Rad Laboratories, goat anti-Mouse IgG #1706516 and anti-rabbit IgG #1706515) in TBS-T for 1 h at room temperature. After the final washes, the proteins were detected by enhanced chemiluminescence. The bands were quantified using Quantity One® 1-D Analysis Software (Bio-Rad Laboratories) and values were normalized with respect to actin. The values were then expressed as a percentage relative to the sham level of OD. The antibodies used were as follows: anti-LC3 (L8918) from Sigma-Aldrich (1:1000), anti-Beclin 1 (Ab62472) from Abcam (1:800), anti-p62/SQSTM1 (P0067) from Sigma-Aldrich, anti-β III tubulin (1637) from Millipore (1:400), goat anti-mouse IgG-HRP (sc-2005) from Santa Cruz Biotechnology (1:10000) and goat anti-rabbit IgG-HRP (sc-2004) from Santa Cruz Biotechnology (1:10,000).

### In vitro experiments

#### SMA animals and spinal cord MN isolation and culture

Severe SMA mice FVB.Cg-Tg(SMN2)^89Ahmb^Smn1^tm1Msd^/J were kindly provided by Dr. J. Esquerda (IRBLLEIDA-Universitat de Lleida). Heterozygous animals were crossed to obtain homozygous *Smn* ^−/−^;SMN2^+/+^ (mutSMA). Littermates mtSMA and *Smn* ^+/+^;SMN2^+/+^ (WT) were used for the experiments. For MNs purification E13 embryos were removed from the uterus and a piece was snipped from the head for genotyping. The REDExtract-N-Amp Tissue PCR Kit (Sigma-Aldrich) was used for genomic DNA extraction and polymerase chain reaction setup, with the following primers: WT forward 5′-CTCCGGATATTGGGATTG-3′, SMA reverse 5′-GGTAACGCCAGGGTTTTCC-3′ and WT reverse 5’-TTTCTTCTGGCTGTGCCTTT-3′. After genotyping, MNs from WT and mutSMA were purified and cultured. Six days after plating, cells were treated with 5 mm of 3-MA. Twelve hours later, protein extracts were obtained and submitted to WB analysis using anti-LC3-II, anti-Smn, and anti-p62/SQSTM1 antibodies.

### WB analysis of cultured MNs

WB analysis was performed as described^70^. After treatment cells were rinsed in ice-cold PBS (pH 7.2). Total cell lysates were collected and resolved in SDS-polyacrylamide gels and transferred onto polyvinylidene difluoride Immobilon-*P* transfer membrane filters (Millipore), using an Amersham Biosciences (Piscataway) semidry Trans-Blot according to the manufacturer’s instructions. The membranes were blotted with the specific antibodies anti-Smn (1:5000) from BD Transduction Laboratories, anti-p62/SQSTM1 antibody (1:1000), and anti-LC3 antibody (1:1000) from Cell Signaling Technology following the instructions of the providers. Unless stated otherwise, to control the specific protein content per lane, membranes were re-probed with a monoclonal anti-α-Tubulin (Tub) antibody (1:50,000, Sigma-Aldrich), as described by the provider. Blots were developed using the Super Signal chemiluminescent substrate (Pierce, Rockford) or the ECL Advance WB detection kit (Amersham Biosciences).

### Statistical analyses

Survival analysis was performed by Kaplan−Meier analysis. All the experiments were performed at least three times and the data were expressed as mean±standard deviation (SD) or standard error (SEM) of the mean. Data were analyzed using one-way ANOVA (for the in vivo* results*) and Student *t*-test (for the in vitro* results*): *p*-values < 0.05 were considered significant.

## Supplementary information

Supplement Figure 1
Supplement Figure Legend
