# Supplementary figures and images for: Inhibition of autophagy delays motoneuron degeneration and extends lifespan in a mouse model of spinal muscular atrophy
Source: Cell Death Dis. 2017 Dec 20;8(12):3223. doi: 10.1038/s41419-017-0086-4 (PMC5870600; doi:10.1038/s41419-017-0086-4)

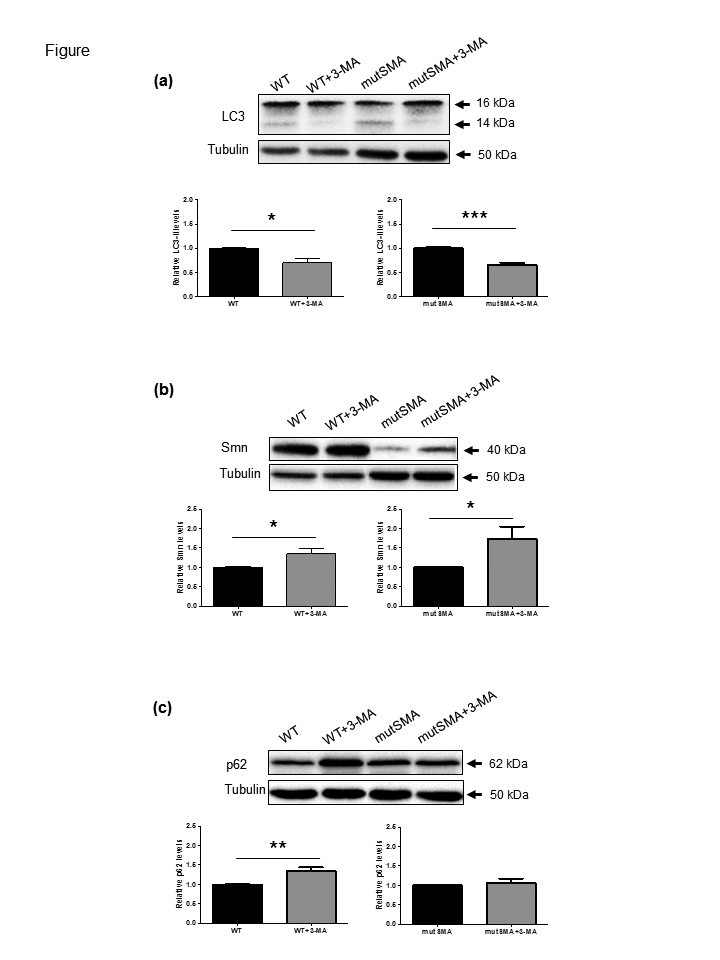

Supplement: Supplementary file 1 — Supplement Figure 1 [file 41419_2017_86_MOESM1_ESM.tif]
