# Supplementary material for: Inhibition of autophagy delays motoneuron degeneration and extends lifespan in a mouse model of spinal muscular atrophy
Source: Cell Death Dis. 2017 Dec 20;8(12):3223. doi: 10.1038/s41419-017-0086-4 (PMC5870600; doi:10.1038/s41419-017-0086-4)
Supplement: Supplementary file 2 — Supplement Figure Legend [file 41419_2017_86_MOESM2_ESM.docx]

**Supplementary Figure Legend**

**SF. 1** 3-MA treatment increases Smn protein level in SMA cultured mice MNs. **(a-c)** Cell lysates were obtained from 6-days WT and mutSMA cultured MNs after 12 hr of 5 mM 3-MA treatment. Protein extracts were probed with anti-LC3-II (**a**), anti-Smn (**b**) and anti-p62/SQSTM1 (**c**) antibodies by WB analysis. Membranes were reprobed with an antibody against Tubulin as a loading control. Graphs values represent the expression of LC3-II, Smn or p62/SQSTM1 versus Tubulin, corresponding to the quantification of three independent experiments±SEM Asterisk indicate significant differences using Student *t*-test (**p*<0.05, ***p*<0.005 and ****p*<0.001).
